# Factors Associated With Uptake of Iron Supplement During Pregnancy Among Women of Reproductive Age in Somaliland: A Cross‐Sectional Study

**DOI:** 10.1002/puh2.70427

**Published:** 2026-10-14

**Authors:** Awo Mohamed Kahie, Mohamed Said Hassan, Suhaib Mohamed Kahie, Abdikadir Abdillahi Husein, Asma Abdirahman

**Affiliations:** ^1^ College of Health Sciences School of Medicine and Surgery Amoud University Borama Somalia; ^2^ School of Postgraduate Studies and Research Amoud University, Amoud Valley Borama Somalia

**Keywords:** anemia, antenatal care, DHS, iron supplementation, maternal health, multilevel analysis, Somaliland

## Abstract

**Background:**

Anemia in pregnancy is a severe public health problem in low‐ and middle‐income countries, yet the uptake of recommended iron supplementation remains critically low. In a post‐conflict and resource‐constrained setting like Somaliland, there is a significant evidence gap regarding the factors that determine whether pregnant women use these essential supplements. This study aimed to identify the individual and community‐level factors associated with the uptake of iron supplements during pregnancy in Somaliland.

**Methods:**

We conducted a cross‐sectional analysis of data from the 2020 Somaliland Demographic and Health Survey (SLDHS), including a nationally representative weighted sample of 2577 women aged 15–49 years who had a live birth in the 5 years preceding the survey. A multilevel binary logistic regression model was used to identify determinants of iron supplement uptake while accounting for the hierarchical data structure.

**Results:**

The uptake of iron supplements was alarmingly low, with only 37.08% of women reporting use during their most recent pregnancy. The strongest predictor was attending four or more antenatal care (ANC) visits (adjusted odds ratio [AOR] = 6.20; 95% confidence interval [CI]: 4.70–8.19). Other significant positive determinants included belonging to a rich household (AOR = 1.93; 95% CI: 1.43–2.60), having a husband ***in paid employment*** (AOR = 2.13; 95% CI: 1.71–2.66), and exposure to television. Conversely, women whose husbands had no formal education had significantly lower odds of uptake (AOR = 0.68; 95% CI: 0.51–0.91). Significant regional disparities were observed, with markedly lower uptake in the Sool and Sanaag regions.

**Conclusion:**

Iron supplement uptake in Somaliland is critically deficient and driven by profound inequities in healthcare access and socioeconomic status. A multipronged strategy is urgently needed, focusing on strengthening ANC services, leveraging mass media and mHealth for targeted health promotion, addressing socioeconomic barriers, and engaging male partners to improve maternal and child health outcomes.

## Graphical abstract

Only 37.08% of pregnant women in Somaliland use iron supplements, with ≥4 ANC visits being the strongest predictor of uptake (AOR = 6.20). Addressing socioeconomic barriers and regional inequities while strengthening ANC services are essential strategies to improve maternal health outcomes in this resource‐constrained setting.

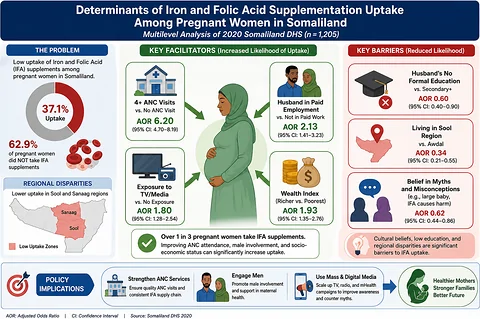

## 1 Introduction

Anemia during pregnancy remains a formidable global public health challenge, exerting a profound toll on maternal and infant health. The World Health Organization (WHO) estimates that nearly 40% of pregnant women worldwide are anemic, a figure that masks stark disparities, with the highest prevalence concentrated in low‐ and middle‐income countries (LMICs) [1]. Iron deficiency is the predominant cause, fueling a cascade of adverse outcomes, including an elevated risk of maternal mortality, preterm birth, low birth weight, and long‐term impaired cognitive development in children [2, 3].

In Somaliland, the 2020 Somaliland Demographic and Health Survey (SLDHS) indicates that anemia remains a significant burden, with approximately 31% of women of reproductive age suffering from some degree of anemia. Despite its prevalence, the clinical outcomes of pregnancies in this region are often compromised by poor nutritional status and a lack of preventive care. The disproportionate burden in LMICs is driven by a convergence of factors, including inadequate dietary iron intake, high rates of infectious diseases, and fragile health systems, unable to deliver essential preventive care consistently.

In response, the WHO advocates for daily oral iron and folic acid supplementation (IFAS) as a core, cost‐effective intervention throughout pregnancy (World Health Organization, 2016) [4]. However, translating this global recommendation into effective local practice is fraught with challenges. Across sub‐Saharan Africa, uptake and adherence to IFAS are critically low, often failing to reach impactful coverage levels [5, 6, 7]. The barriers are complex and interwoven, spanning individual factors, like socioeconomic status and educational level, health system factors, such as the accessibility and quality of antenatal care (ANC), and sociocultural factors, including local beliefs and awareness of the supplement's benefits [8].

Somaliland, located in the Horn of Africa, represents a context of unique vulnerability. Decades of instability have resulted in a fragmented health system with limited public funding, high maternal mortality rates, and significant barriers to healthcare access, particularly for its large rural and nomadic populations [9]. The health system in Somaliland follows a tiered structure where the Essential Package of Health Services (EPHS) mandate the provision of IFAS free of charge at all public health facilities. However, supply‐side issues, such as frequent stock‐outs of supplements and the uneven distribution of health personnel, mean that an ANC visit does not always guarantee that a woman will physically receive the supplements. Furthermore, even when provided, systemic gaps in counseling and patient education often leave women unaware of the correct dosage or the importance of oral consumption [7]. Although IFAS is officially part of the essential health services package, a critical evidence gap exists regarding its actual implementation and use. Investigating these factors is essential for designing targeted, context‐specific interventions that can effectively improve maternal nutrition and health outcomes.

## 2 Methods and Materials

### 2.1 Study Area

The research was conducted in Somaliland, officially known as the Republic of Somaliland, a self‐declared sovereign entity in the Horn of Africa. This region comprises six geographical areas: Awdal, Marodijeh, Sahil, Togdheer, Sanaag, and Sool. Access to healthcare services poses significant challenges, particularly in rural communities. To address these issues, the Ministry of Health Development has implemented the EPHS, aligned with the WHO's foundational health system building blocks. Healthcare delivery in Somaliland is structured into five tiers, from community‐level preventive care to regional hospitals for complex cases [5].

### 2.2 Study Design and Study Period

This study used a cross‐sectional design, analyzing data from the SLDHS, which was carried out nationally in 2020. Its aim was to investigate the factors contributing to the uptake of iron supplements among pregnant women.

### 2.3 Sample Size and Sampling

A total of 2577 women aged 15–49 years were included in the study, drawn from the SLDHS dataset and weighted for analysis. The sampling strategy considered six geographic regions and various types of residences: urban, rural, and nomadic. A multistage stratified cluster sampling technique was employed to ensure a representative sample [5].

### 2.4 Study Variables

The outcome variable investigated in this study was iron supplementation during pregnancy, classified as “Yes” or “No.” Respondents in the SLDHS were prompted to indicate whether they had received any iron tablets or syrup during their most recent pregnancy. The response was coded as “Yes = 1” and “No = 0.”

The independent variables were classified into two levels as follows:

- **Community‐Level Variables**: These included region and type of residence (rural and urban).
- **Individual‐Level Variables**: These included: number of ANC visits (<4 visits vs. ≥4 visits), maternal age, maternal education, husband's education, household wealth index (poor, middle, and rich), ownership of a mobile telephone, internet usage, frequency of watching television, women's occupation *(categorized as “engaged in paid employment” or “no paid employment” to acknowledge that women without formal jobs still perform significant domestic work)*, and husband's work status *(engaged in paid employment vs. no paid employment)*.

### 2.5 Data Management and Analysis

Data were managed and analyzed using STATA version 16.0. The svy command was used to account for the complex survey design (weighting, clustering, and stratification). Descriptive statistics (weighted frequencies and percentages) were used to summarize participant characteristics. At the bivariate level, the Pearson chi‐square test was used to assess associations between each independent variable and iron supplement uptake.

Given the hierarchical data structure (individuals nested within communities), a multilevel binary logistic regression model was used to identify determinants of iron supplement uptake. A three‐model building approach was utilized as follows:

- **Model I (Null Model)**: An empty model with no predictors to assess community‐level variance via the intraclass correlation coefficient (ICC).
- **Model II (Individual‐Level Model)**: Included only individual and household‐level variables.
- **Model III (Full Model)**: Included both individual‐ and community‐level variables. Model fit was assessed using the Akaike information criterion (AIC) and Bayesian information criterion (BIC). Results are presented as adjusted odds ratios (AORs) with 95% confidence intervals (CIs).

## 3 Results

### 3.1 Descriptive Characteristics of the Study Population

Table 1 presents a comprehensive descriptive analysis of a weighted sample of 2577. Geographically, the population is diverse, with Marodijeh (27.59%) and Togdheer (26.29%) being the most represented regions. A majority (55.12%) resides in rural areas, and a substantial 80.51% of women report fewer than the recommended four ANC visits. Crucially, data from the SLDHS report highlight that among the small percentage of women who do receive iron, many do not adhere to the full 90+ day course, and approximately 1 in 10 births in the region are categorized as low birth weight, likely a downstream effect of the low IFAS uptake and high maternal anemia rates.

**TABLE 1:** Descriptive characteristics of the study population (*N* = 2577).

Variables	Categories	Weighted frequency (*n*)	Percentage
**Community‐level factors**			
Region	Awdal	245	9.50
	Marodijeh	711	27.59
	Sahil	146	5.66
	Togdheer	677	26.29
	Sool	357	13.85
	Sanaag	441	17.11
Type of place of residence	Rural	1420	55.12
	Urban	1157	44.88
**Individual‐level factors**			
**Number of antenatal visits during pregnancy**	<4	2075	80.51
	≥4	502	19.49
Maternal educational level	No education	2020	78.40
	Primary	400	15.50
	Secondary	99	3.84
	Higher	58	2.26
Frequency of watching television	At least once a week	480	18.62
	Less than once a week	79	3.08
	Not at all	2018	78.30
Owns a mobile telephone	Yes	1960	76.08
	No	617	23.92
Ever used internet	Yes	294	11.40
	No	2283	88.60
Wealth index combined	Poor	1236	47.96
	Middle	278	10.80
	Rich	1063	41.24
Maternal age	15–29 years	1286	49.90
	30–39 years	1052	40.82
	40–49 years	239	9.28
Husband ever attended school	Yes	295	11.42
	No	2282	88.58
Women's occupation	No paid employment	2555	99.17
	In paid employment	22	0.83
Total children ever born	0–1	327	12.70
	2–3	764	29.66
	4–5	669	25.96
	6–7	436	16.92
	8–9	246	9.52
	10+	135	5.24
Husband worked	No paid employment	1293	50.18
	In paid employment	1284	49.82

Economic vulnerability is pronounced, with nearly half (47.96%) classified as poor and a striking 99.17% of women not in paid employment, further exacerbated by 50.18% of husbands/partners not being in paid employment in the past 12 months.

### 3.2 Prevalence of Uptake of Iron Supplement

Figure 1, illustrating the weighted uptake of iron supplements during pregnancy among women of reproductive age in Somaliland, reveals a significant gap in adherence to recommended ANC practices. The data indicate that only 37.08% of pregnant women reported taking iron supplements, whereas a substantial majority (62.92%) did not.

**FIGURE 1:**
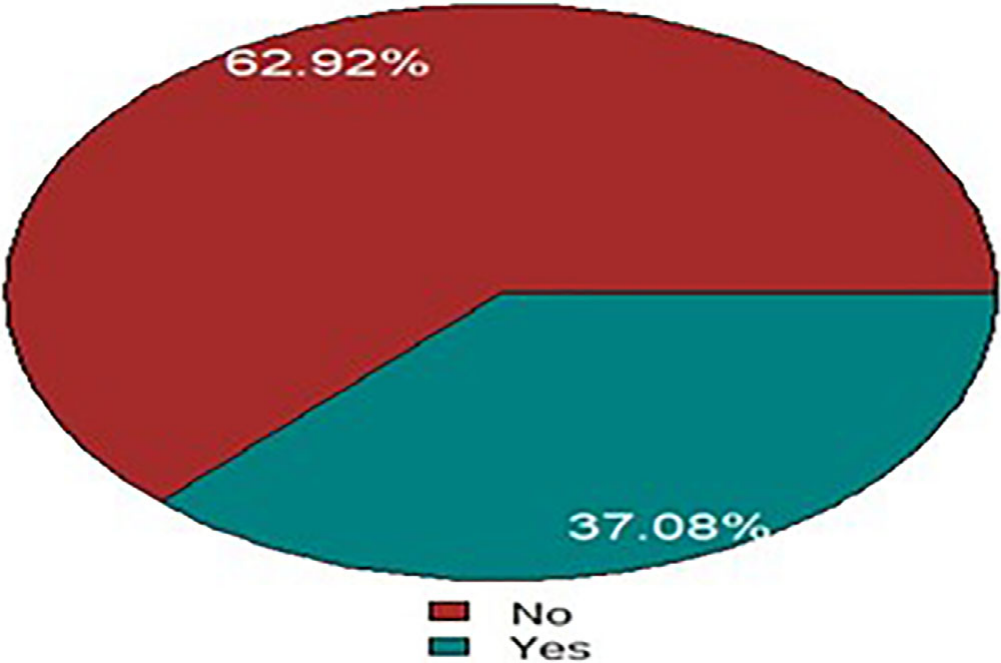
The prevalence of uptake of iron supplement during pregnancy among women of reproductive age in Somaliland based on SLDHS.

### 3.3 Bivariate Analysis of Factors Associated With Iron Supplement Uptake

The bivariate analysis presented in Table 2 shows that several factors were significantly associated with iron supplement uptake. At the community level, a strong association was observed between geographical region and uptake (*χ* ^2^ = 227.51, *p* < 0.00001), with Sool and Sanaag showing substantially lower rates compared to Marodijeh and Togdheer. Conversely, no significant association was found with place of residence (*χ* ^2^ = 2.70, *p* = 0.4745). The number of antenatal visits showed a highly significant association (*χ* ^2^ = 361.47, *p* < 0.00001), with women attending ≥4 visits more likely to report uptake compared to those with <4 visits. At the individual level, maternal age did not show a significant association (*χ* ^2^ = 10.13, *p* = 0.1380), though mothers aged 40–49 years had a lower uptake rate. However, a significant association was found between maternal educational level and uptake (*χ* ^2^ = 153.12, *p* < 0.00001), with uptake rising from those with no education to secondary education. Similarly, the frequency of watching television (*χ* ^2^ = 160.40, *p* < 0.00001) and mobile telephone ownership (*χ* ^2^ = 57.32, *p* < 0.00001) were both highly significant, with those watching TV weekly and owning a mobile phone more likely to report uptake. Internet use also showed a strong association (*χ* ^2^ = 111.33, *p* < 0.00001), as did the husband's employment status (*χ* ^2^ = 290.02, *p* < 0.00001), with higher uptake reported when the husband worked. The wealth index indicated a significant relationship (*χ* ^2^ = 345.19, *p* < 0.00001), showing a clear dose–response correlation with uptake increasing from the poor to the rich. Additionally, a significant association was found regarding whether the husband ever attended school (*χ* ^2^ = 16.03, *p* = 0.0143), whereas women's occupation did not show a significant association (*χ* ^2^ = 1.91, *p* = 0.4918). Finally, the total number of children ever born showed a statistically *nonsignificant* association with iron supplement uptake (*χ* ^2^ = 19.63, *p* = 0.1113).

**TABLE 2:** Bivariate analysis of factors associated with uptake of iron supplement during pregnancy.

Variable	Categories	No, *n* (%)	Yes, *n* (%)	*χ* ^2^ (*p* value)
**Community‐level factors**				
Region	Awdal	152 (62.15)	93 (37.85)	227.51 (*p* < 0.0001)
	Marodijeh	367 (51.61)	344 (48.39)	
	Sahil	91 (62.62)	55 (37.38)	
	Togdheer	349 (51.56)	328 (48.44)	
	Sool	303 (84.85)	54 (15.15)	
	Sanaag	359 (81.37)	82 (18.63)	
Place of residence	Rural	874 (61.55)	546 (38.45)	2.70 (*p* = 0.4745)
	Urban	747 (64.58)	410 (35.42)	
**Individual‐level factors**				
**Number of antenatal visits**	<4	1485 (71.56)	590 (28.44)	361.47 (*p* < 0.0001)
	≥4	137 (27.22)	365 (72.78)	
Maternal age	15–29 years	786 (61.14)	500 (38.86)	10.13 (*p* = 0.1380)
	30–39 years	664 (63.11)	388 (36.89)	
	40–49 years	171 (71.64)	68 (28.36)	
Maternal educational level	No education	1385 (68.56)	635 (31.44)	153.12 (*p* < 0.0001)
	Primary	190 (47.5)	210 (52.5)	
	Secondary	28 (28.65)	71 (71.35)	
	Higher	17 (30.00)	41 (70.00)	
Frequency of watching television	At least once a week	187 (38.98)	293 (61.02)	160.40 (*p* < 0.0001)
	Less than once a week	43 (54.96)	36 (45.04)	
	Not at all	1391 (68.92)	627 (31.08)	
Owns a mobile telephone	Yes	1156 (59.00)	804 (41.00)	57.32 (*p* < 0.0001)
	No	465 (75.39)	152 (24.61)	
Ever used internet	Yes	105 (35.74)	189 (64.26)	111.33 (*p* < 0.0001)
	No	1519 (66.41)	764 (33.59)	
Husband worked	No paid employment	1018 (78.71)	275 (21.29)	290.02 (*p* < 0.0001)
	In paid employment	606 (47.24)	678 (52.76)	
Wealth index combined	Poor	1025 (82.88)	211 (17.12)	345.19 (*p* < 0.0001)
	Middle	165 (59.47)	113 (40.53)	
	Rich	495 (46.52)	568 (53.48)	
Husband ever attended school	Yes	156 (52.8)	139 (47.2)	16.03 (*p* = 0.0143)
	No	1466 (64.25)	816 (35.75)	
Women's occupation	No paid employment	1609 (63.03)	946 (36.97)	1.91 (*p* = 0.4918)
	In paid employment	10 (44.7)	12 (55.3)	
Total children ever born	0–1	182 (55.74)	145 (44.26)	19.63 (*p* = 0.1113)
	2–3	468 (61.25)	296 (38.75)	
	4–5	440 (65.75)	229 (34.25)	
	6–7	273 (62.55)	163 (37.45)	
	8–9	158 (64.20)	88 (35.80)	
	10+	101 (74.57)	34 (25.43)	

### 3.4 Multilevel Analysis of Factors Associated With the Uptake of Iron Supplements

Table 3 presents the results of a multilevel analysis investigating individual and community‐level factors associated with the uptake of iron supplements during pregnancy among women of reproductive age in Somaliland. For clarity and consistency with the methods, the results are presented in three progressive models: Model I (null model), Model II (individual‐level model), and Model III (full model).

**TABLE 3:** Multilevel analysis of factors associated with uptake of iron supplement during pregnancy.

Variable	Categories	Model II (individual‐level model) AOR (95% CI)	Model III (full model) AOR (95% CI)
**Community‐level factors**			
Region	Awdal		Ref
	Marodijeh		0.98 (0.50–1.89)
	Sahil		1.45 (0.93–2.28)
	Togdheer		1.07 (0.69–1.66)
	Sool		0.51 (0.33–0.80)^***^
	Sanaag		0.63 (0.40–0.99)^*^
**Individual‐level factors**			
Number of antenatal visits	<4	Ref	Ref
	≥4	5.11 (3.89–6.70)^***^	6.20 (4.70–8.19)^***^
Maternal education	No education	Ref	Ref
	Primary	1.20 (0.91–1.58)	1.30 (0.98–1.74)
	Secondary	1.43 (0.79–2.61)	1.78 (0.96–3.30)
	Higher	1.47 (0.60–3.61)	1.41 (0.55–3.60)
Maternal age	<30 years	Ref	Ref
	30–39 years	0.99 (0.78–1.26)	0.97 (0.76–1.23)
	40–49 years	0.73 (0.49–1.10)	0.77 (0.52–1.15)
Frequency of watching television	At least once a week	Ref	Ref
	Less than once a week	0.51 (0.25–1.03)	0.45 (0.23–0.87)^*^
	Not at all	0.75 (0.53–1.07)	0.60 (0.43–0.85)^**^
Owns mobile telephone	Yes	Ref	Ref
	No	0.73 (0.57–0.93)^**^	0.78 (0.61–0.99)^*^
Internet usage	Yes	Ref	Ref
	No	0.88 (0.57–1.34)	0.76 (0.51–1.15)
Husband work	No	Ref	Ref
	Yes	1.80 (1.43–2.27)^***^	2.13 (1.71–2.66)^***^
Wealth index	Poor	Ref	Ref
	Middle	1.56 (1.12–2.18)^**^	1.36 (0.97–1.91)
	Rich	1.89 (1.44–2.49)^***^	1.93 (1.43–2.60)^***^
Husband ever attended school	Yes	Ref	Ref
	No	0.74 (0.55–1.00)	0.68 (0.51–0.91)^**^
Total children ever born	0–1	Ref	Ref
	2–3	0.86 (0.62–1.20)	0.82 (0.60–1.13)
	4–5	0.82 (0.58–1.17)	0.75 (0.54–1.06)
	6–7	1.00 (0.67–1.49)	0.95 (0.64–1.40)
	8–9	1.12 (0.71–1.77)	0.99 (0.63–1.55)
	10+	0.81 (0.46–1.42)	0.65 (0.37–1.12)

Abbreviation: CI, confidence interval.

*** *p* < 0.001.

** *p* < 0.01.

* *p* < 0.05.

At the community level, significant regional disparities were observed in *Model III*, with women in Sool (AOR = 0.51, 95% CI: 0.33–0.80) and Sanaag (AOR = 0.63, 95% CI: 0.40–0.99) showing significantly lower odds of iron supplement uptake compared to the reference region of Awdal. […] The number of antenatal visits consistently emerged as a highly significant factor across all models, with women attending ≥4 visits having substantially higher odds of uptake (*Model III*: AOR = 6.20, 95% CI: 4.70–8.19) compared to those with fewer than four visits.

Among individual‐level factors, maternal education did not show a statistically significant association with uptake across the models. Similarly, maternal age also did not demonstrate a significant association. However, the frequency of watching television showed a significant inverse relationship in *Model III*, with women watching TV less than once a week (AOR = 0.45, 95% CI: 0.23–0.87) and not at all (AOR = 0.60, 95% CI: 0.43–0.85) having lower odds of uptake compared to those watching at least once a week. *Conversely, not owning a mobile telephone was significantly associated with lower odds of uptake compared to those who did own one* (AOR = 0.78, 95% CI: 0.61–0.99). Internet usage did not show a significant association. Husband's employment status was a highly significant predictor, with women whose husbands worked having considerably higher odds of uptake (*Model III*: AOR = 2.13, 95% CI: 1.71–2.66). The wealth index consistently showed a significant positive association, with women in the rich category demonstrating higher odds of uptake (*Model III*: AOR = 1.93, 95% CI: 1.43–2.60) compared to the poor. *Furthermore, women whose husbands had not attended school had significantly lower odds of uptake compared to those whose husbands had attended* (AOR = 0.68, 95% CI: 0.51–0.91). The total number of children ever born did not show a statistically significant association with iron supplement uptake across any of the models.

### 3.5 Random Effect Analysis and Model Comparison

The random effects analysis, presented in Table 4, employs a mixed‐effects logistic regression to explore how individual and community‐level factors impact the uptake of iron supplements while accounting for data clustering. Model I (the null model), featuring only a random intercept, reveals significant cluster variance (*τ* ^2^ = 2.02, *p* < 0.001) and an ICC of 0.38, indicating that 38% of the variance is attributable to differences between clusters, highlighting the necessity of a multilevel approach. In Model II (the individual‐level model), which incorporates individual‐level predictors, the cluster variance significantly decreases to 0.46 (*p* < 0.001), with an ICC dropping to 0.12, suggesting that individual characteristics explain a substantial amount of the cluster‐level variation. Finally, Model III (the full model), which includes both individual and community predictors, dramatically reduces cluster variance to 0.04 and results in a very low ICC of 0.01, with a nonsignificant *p* value for the LR test (0.190). This indicates that the inclusion of both levels of predictors sufficiently accounts for the clustering in the data. Model comparison through AIC, BIC, and −2Log‐likelihood demonstrates that Model III provides the best fit, confirming its effectiveness in capturing the outcome's complexity.

**TABLE 4:** Random effects analysis and model comparison.

Measurement of variation	Model I (null model)	Model II (individual‐level model)	Model III (full model)
*τ* ^2^ (cluster variance)	2.02	0.46	0.04
*p* value (LR test vs. logistic model)	<0.001	<0.001	0.190
ICC	0.38	0.12	0.01
**Model fit statistics**			
AIC	2972.9	2843.583	2639.904
BIC	2984.727	2961.854	2793.656
−2Log‐likelihood (deviance)	2968.9	2803.583	2587.904

Abbreviations: AIC, Akaike information criterion; BIC, Bayesian information criterion; ICC, intraclass correlation coefficient.

## 4 Discussion

This study provides critical insights into iron supplement uptake in Somaliland, revealing a critically low prevalence and highlighting the powerful roles of healthcare access, socioeconomic status, and media exposure. The finding that only 37.08% of pregnant women took any iron supplements is alarmingly deficient, falling far short of the WHO's 80% coverage target for routine interventions and signaling a major public health failure [1]. This rate is comparable to or lower than those in other challenging settings in sub‐Saharan Africa [7, 10, 11, 12]. Interestingly, although Somaliland struggles with an uptake of 37%, neighboring regions, such as the Ethiopian Somali Region, have reported even lower ANC 4+ attendance and IFAS adherence, suggesting a pervasive cross‐border challenge in providing maternal health services to nomadic and pastoralist populations [13, 14].

The most potent predictor of iron supplement uptake was attending four or more ANC visits. However, it is notable that even among those attending ANC, oral consumption is not guaranteed. Behavioral barriers, such as the unpleasant metallic taste of iron, constipation, and local myths, regarding the supplements making the baby “too large” for delivery, often lead women to receive the pills but not actually ingest them orally. Furthermore, even when women are motivated, supply‐side inconsistencies mean that facilities frequently run out of stock, leaving providers unable to dispense the medication despite the visit.

Socioeconomic factors emerged as powerful determinants. The strong, dose‐dependent association with household wealth and the significant influence of a husband's employment and education align with studies across LMICs [15, 16, 17]. In this study, the wealth index was used as a composite variable; however, the influence of its individual components—such as livestock ownership in nomadic areas versus cash income in urban areas—suggests that economic stability primarily facilitates uptake by reducing the “opportunity cost” of traveling to a clinic. The husband's role is particularly salient in a patriarchal context, like Somaliland. An employed and educated partner is more likely to understand, support, and enable his wife's access to healthcare. This finding, juxtaposed with the fact that 99% of women were not in paid employment, underscores that maternal health is inextricably linked to household economics and male engagement. The term “no paid employment” is used here to reflect that although these women are not earning a salary, they are often engaged in demanding household labor and childcare which limits their time for healthcare visits.

Although this study focused on uptake (whether a woman ever took the supplement), future research must address the varying levels of adherence (the duration and consistency of use), as many women who “take” the supplement do not complete the recommended 90‐day course.

## 5 Strengths and Limitations

The primary strength of this study is its use of a large, nationally representative dataset. However, there are limitations. The cross‐sectional design prevents causal claims. Furthermore, the survey did not capture supply‐side variables, such as facility‐level stock‐outs or the specific quality of counseling provided by midwives, both of which are critical to understanding why uptake remains low even among ANC attendees.

## 6 Conclusion and Recommendations

The uptake of iron supplementation during pregnancy in Somaliland is critically low. On the basis of these findings, the following recommendations are proposed as follows:

1. Strengthen and Promote ANC Services: Prioritize increasing the coverage and quality of ANC. This must include a robust supply chain management system to ensure that iron supplements are consistently in stock so that an ANC visit always results in the provision of the medication.
2. Implement Targeted and Accessible Health Promotion: Design public health campaigns to raise awareness. These campaigns should specifically address side‐effect management and debunk myths regarding oral iron to bridge the gap between receiving the supplement and actually consuming it.
3. Address Socioeconomic Disparities: Integrate maternal nutrition programs with poverty reduction.
4. Engage Male Partners: Develop health education initiatives that actively involve husbands to foster a supportive environment for maternal health and encourage their participation in ANC and the financial support of nutritional adherence.

## Author Contributions

Awo Mohamed Kahie conceptualized the study, performed the data analysis, interpreted the results, and wrote the original draft of the manuscript. Mohamed Said Hassan contributed to the study design, methodology, and reviewed the manuscript for intellectual content. Suhaib Mohamed Kahie assisted with data curation, validation of the analysis, and reviewed the final manuscript. Abdikadir Abdillahi Husein contributed to the literature review, interpretation of findings, and critical revision of the manuscript. Asma Abdirahman provided supervision, contributed to the discussion section, and critically reviewed the manuscript. All authors read and approved the final manuscript.

## Funding

The authors have nothing to report.

## Ethics Statement

This study is a secondary analysis of publicly available, anonymized data from the 2020 Somaliland Health and Demographic Survey (SHDS). The original SHDS survey protocol was approved by the Ministry of Planning and National Development of the Somaliland Government and the ICF Institutional Review Board in the United States.

## Consent

Informed consent was obtained from all participants (or their legal guardians for children) before the original data collection. Accordingly, no further ethical approval was required for this secondary analysis.

## Conflicts of Interest

The authors declare no conflicts of interest.

## Transparency Statement

“**Mohamed Said Hassan** affirms that this manuscript is an honest, accurate, and transparent account of the study being reported; that no important aspects of the study have been omitted; and that any discrepancies from the study as planned (and, if relevant, registered) have been explained.”

## Data Availability

The data that support the findings of this study are available from the corresponding author upon reasonable request.

## References

[1] WHO , WHO Global Anaemia Estimates, Edition (WHO, 2025), https://www.who.int/data/gho/data/themes/topics/anaemia_in_women_and_children.

[2] G. A. Stevens , M. M. Finucane , L. M. De‐Regil , et al., “Global, Regional, and National Trends in Haemoglobin Concentration and Prevalence of Total and Severe Anaemia in Children and Pregnant and Non‐Pregnant Women for 1995–2011: A Systematic Analysis of Population‐Representative Data,” Lancet Global Health 1, no. 1 (2013): e16–e25, 10.1016/s2214-109x(13)70001-9. PMC4547326 25103581

[3] A. O. Ibrahim , B. A. Daud , W. S. Ahmed , et al., “Determinants of Newborn Birth Size in Somaliland: An Ordinal Logistic Regression Analysis,” Archives of Public Health 83, no. 1 (2025): 315. 10.1186/s13690-025-01765-3 PMC12752441 41462334

[4] World Health Organization . WHO Recommendations on Antenatal Care for a Positive Pregnancy Experience (World Health Organization, 2016). ISBN: 978‐92‐4‐154991‐2. 28079998

[5] T. Gensa Geta , S. A. Abdiwali , and M. Farah , “Magnitude and Factors Associated With Teenage Pregnancy in Somaliland: Evidence From Somaliland Health and Demographic Survey, 2020,” International Journal of Women's Health 15 (2023): 1443–1452, 10.2147/ijwh.s410489. PMC10505387 37724308

[6] J. P. Wirth , F. Sesay , J. Mbai , et al., “Risk Factors of Anaemia and Iron Deficiency in Somali Children and Women: Findings From the 2019 Somalia Micronutrient Survey,” Maternal & Child Nutrition 18, no. 1 (2022): e13254. 10.1111/mcn.13254 PMC8710091 34405549

[7] A. I. Mohamed , J. Mohamed , M. M. Abdillahi , B. A. Abdeeq , and T. B. Lema , “Magnitude and Determinants of Adherence to Iron‐Folic Acid Supplementation Among Somaliland Pregnant Women in Ahmed‐Dhagah District: A Facility Based Cross‐Sectional Study,” Clinical Epidemiology and Global Health 26 (2024): 101565.

[8] F. W. Sendeku , G. G. Azeze , and S. L. Fenta , “Adherence to Iron‐Folic Acid Supplementation Among Pregnant Women in Ethiopia: A Systematic Review and Meta‐Analysis,” BMC Pregnancy Childbirth 20, no. 1 (2020): 138, 10.1186/s12884-020-2835-0. PMC7057669 32131751

[9] SLHDS , The Somaliland Health and Demographic Survey 2020 (Central Statistics Department, Ministry of Planning and National Development, Somaliland Government, 2020), https://www.somalilandcsd.org/somaliland‐health‐and‐demography‐survey‐slhd2020/.

[10] A. Titilayo , M. E. Palamuleni , and O. Omisakin , “Sociodemographic Factors Influencing Adherence to Antenatal Iron Supplementation Recommendations Among Pregnant Women in Malawi: Analysis of Data From the 2010 Malawi Demographic and Health Survey,” Malawi Medical Journal 28, no. 1 (2016): 1–5, 10.4314/mmj.v28i1.1. PMC4864384 27217909

[11] T. G. Demie , G. T. Gessese , B. T. Woldeamanuel , T. D. Biratu , and S. Handebo , “Adherence to Iron Supplement Intake During Pregnancy and Associated Factors in Ethiopia: Further Analysis of a National Population‐Based Study,” Food Science & Nutrition 11, no. 9 (2023): 5460–5471. 10.1002/fsn3.3503 PMC10494606 37701196

[12] A. E. Yismaw , H. B. Tulu , F. Y. Kassie , and B. M. Araya , “Iron‐Folic Acid Adherence and Associated Factors Among Pregnant Women Attending Antenatal Care at Metema District, Northwest Ethiopia,” Frontiers in Public Health 10 (2022): 978084. 10.3389/fpubh.2022.978084 PMC9716073 36466514

[13] M. M. Abdilahi , J. Kiruja , B. O. Farah , et al., “Prevalence of Anemia and Associated Factors Among Pregnant Women at Hargeisa Group Hospital, Somaliland,” BMC Pregnancy Childbirth 24, no. 1 (2024): 332. 10.1186/s12884-024-06539-3 PMC11080199 38724919

[14] A. Y. Nuh , “Residence Inequality of Maternal Health Service Utilization Among Women in Somaliland: An Analysis From the Somaliland Demographic Health Survey in 2020,” Journal of Comprehensive Health 13 (2025): 89–95, 10.25259/jch_59_2024.

[15] B. Genet , Adherence to Iron Folate and Associated Factors Among Pregnant Women Attending Antenatal Clinic in Holeta Town Public Health Facilities , West Shewa, Oromia, Ethiopia 2024 (Doctoral dissertation, Ambo University).

[16] A. A. Mohamed , S. A. Ali , A. I. Yasin , et al., “Determinants of Deworming During Pregnancy in Somaliland: A Binary and Multivariate Logistic Regression Analysis,” Discover Social Science and Health 5, no. 1 (2025): 1–8.

[17] L. Kessani , R. Kumar , A. Rathore , and R. Khalid , “Associated Factors and Compliance With Iron‐Folic Acid Therapy Among Pregnant Women of Karachi, Pakistan,” Rawal Medical Journal 43, no. 2 (2018): 319.

